# Non-iterative geometric approach for inverse kinematics of redundant lead-module in a radiosurgical snake-like robot

**DOI:** 10.1186/s12938-017-0383-2

**Published:** 2017-08-01

**Authors:** Olatunji Mumini Omisore, Shipeng Han, Lingxue Ren, Nannan Zhang, Kamen Ivanov, Ahmed Elazab, Lei Wang

**Affiliations:** 1 Research Centre for Medical Robotics and Minimally Invasive Surgical Devices, Shenzhen Institutes of Advanced Technology, Chinese Academy of Sciences, Shenzhen, 518055 China; 2 Shenzhen College of Advanced Technology, University of Chinese Academy of Sciences, Shenzhen, China; 3 Department of Computer Science, Misr Higher Institute for Commerce and Computers, Mansoura, Egypt

**Keywords:** Medical robotics, Inverse kinematics, Geometric method, Serial link manipulator, Modular snake robots, Joint angles

## Abstract

**Background:**

Snake-like robot is an emerging form of serial-link manipulator with the morphologic design of biological snakes. The redundant robot can be used to assist medical experts in accessing internal organs with minimal or no invasion. Several snake-like robotic designs have been proposed for minimal invasive surgery, however, the few that were developed are yet to be fully explored for clinical procedures. This is due to lack of capability for full-fledged spatial navigation. In rare cases where such snake-like designs are spatially flexible, there exists no inverse kinematics (IK) solution with both precise control and fast response.

**Methods:**

In this study, we proposed a non-iterative geometric method for solving IK of lead-module of a snake-like robot designed for therapy or ablation of abdominal tumors. The proposed method is aimed at providing accurate and fast IK solution for given target points in the robot’s workspace. *n*-*1* virtual points (VPs) were geometrically computed and set as coordinates of intermediary joints in an *n*-link module. Suitable joint angles that can place the end-effector at given target points were then computed by vectorizing coordinates of the VPs, in addition to coordinates of the base point, target point, and tip of the first link in its default pose. The proposed method is applied to solve IK of two-link and redundant four-link modules.

**Results:**

Both two-link and four-link modules were simulated with Robotics Toolbox in Matlab 8.3 (R2014a). Implementation result shows that the proposed method can solve IK of the spatially flexible robot with minimal error values. Furthermore, analyses of results from both modules show that the geometric method can reach 99.21 and 88.61% of points in their workspaces, respectively, with an error threshold of 1 mm. The proposed method is non-iterative and has a maximum execution time of 0.009 s.

**Conclusions:**

This paper focuses on solving IK problem of a spatially flexible robot which is part of a developmental project for abdominal surgery through minimal invasion or natural orifices. The study showed that the proposed geometric method can resolve IK of the snake-like robot with negligible error offset. Evaluation against well-known methods shows that the proposed method can reach several points in the robot’s workspace with high accuracy and shorter computational time, simultaneously.

## Background

The impact of robotic technology has brought about advancements in different facets of life such as automobile assembly, military assignments, and agricultural sector. Alongside, incorporating the technology into medical practice plays a significant role in diagnosis and therapy of many medical conditions [1]. Robotics is rapidly becoming useful in interventional surgery because human experts have limited views while carrying out operations on internal organs with minimal or no invasion. However, robotic arms can be designed to move towards targeted organs in a patient and precisely irradiate selected area of tissue that contains tumor from a given position.

Despite the vigorous attention received by surgical robots in the last three decades, real-time applications of highly articulated robots for surgical operations are still at infancy [2]. Usually, robotic manipulators are made by a sequence of serial, parallel, or hybrid links that extend from fixed base to the end-effector. The links are connected to one another by some joint mechanisms for flexible control to allow surgeons to access intended internal organs [3, 4]. Investigation of internal organs through natural orifices or single-port incision has given rise to the development of snake robots. In practice, such robots correspond to serial-link manipulators with a high level of redundancy for spatial navigations. This flexible nature has given more preferences to serial-link manipulators over their parallel counterparts in medical intervention [5]. In minimally invasive surgery (MIS), redundant serial-link manipulators can be controlled to imitate serpentine postures; thus, exhibits varying navigational patterns useful in complex environments [6].

Conventionally, a serial-link robot requires six degrees of freedom (DoF) to reach manipulated objects in arbitrary position and orientation of a robot’s workspace. However, having more-than-needed degrees to perform given tasks makes it dexterous; hence, advantageous in optimal control, path analysis, and obstacle avoidance. Despite these merits in interventional procedures, the performance of snake-like robot is limited by the complexity involved in solving its kinematics, which is distinguished into forward and inverse mappings [7]. Kinematics refers to the representation of the end-effector’s pose in Cartesian or joint space. Forward kinematics is to determine the location of end effector in Cartesian space by direct substitution of link parameters into Denavit–Hartenberg (DH) transformation matrices [8, 9]. On the other hand, inverse kinematics (IK) solutions are essential to determining appropriate joint angles required to drive a robot’s end-effector to given target points during medical procedures.

In Pisla et al. [10], geometric and kinematic models were proposed for positioning active tool-tip instruments fixed at distal part of a surgical hybrid robot. Tool-tip instruments such as surgical blades, radioactive source, and camera; plays important roles in master–slave teleoperated system used in surgical robotics. The intuitive system, da Vinci surgical robot, is a foremost system designed for a range of surgical tasks such as cardiothoracic and gastrointestinal procedures. The robotic platform has motivated several research works in different areas of medical robotics, specifically surgical tasks. For instance, Ota et al. [11] developed a highly articulated surgical robot for intra-pericardial therapy with minimal invasion. Also, Simaan et al. [12] developed a three-armed robotic system for MIS of throat. Each module consists of two DoF snake-like unit and a detachable parallel manipulator. However, the shape memory alloy actuation, adopted for control, posed great limitations in terms of response time and accuracy which are the major metrics used for evaluation of surgical robots. Dupont et al. [13] proposed determining the pose of highly articulated robot constituted by concentrically combined pre-curved elastic tubes. However, complexity of the kinematic model increased with the number tubal segments and thus affects positioning accuracy of the robot. Recently, several laboratories have proposed different designs of snake-like robots for surgical and radiotherapeutic procedures [14–17].

Many algorithmic solutions have been proposed for solving IK of serial-link manipulators with high DoF. Generally, these algorithms can be categorized as analytical or numerical approaches. Analytical approach is a closed-form solution of IK obtained from pure algebra and algebraic geometry. Both methods have been applied to solve IK problems in serial-link redundant robots. In an extensive study, Chirkijian [18] presented a novel framework for modeling IK of hyper redundant robots which are analogous to snake in morphology with planar rotational axis. Also, Sheng et al. proposed a geometric method for solving joint angles in a hyper-redundant manipulator [19]. Theoretic analysis and simulation results show that the method can precisely obtain accurate joint angles required by the manipulator’s end effector to reach a given target in the joint space with fewer computations. However, this approach is limited to planar robots. The geometric method can be extended by spatial decomposition of the robot’s workspace into multiple independent planes. Thence, IK of a robot’s links can be solved by exploring the individual links in their respective planes. In an attempt for 3D path planning, Yahya et al. [20] presented a unique-angle geometric method for positioning the end-effector of planar redundant manipulators in the workspace. The method was adapted for 3D manipulator with a twisted joint at the first joint, while a unique value was determined for other joints. The solution makes the manipulator to always exhibit open polygonal shapes which cannot be suitable for operations in confined environments.

Meanwhile, it is important to consider IK solutions for highly articulated robots that are capable of spatial navigation. These type of manipulators can enhance dexterity in MIS. Recently, geometric approaches have appeared as a better way to solving IK of robotic manipulators as noted in [20–22]. These studies proposed geometrical models for IK of hyper redundant manipulators which could be used to assist in interventional procedures. To find the optimum solution in 3D workspace, geometric models in these studies can solve n-link redundant manipulator having the first link connected to a fixed base with twisted joint. Conclusively, application of closed-form solutions involves the use of transcendental trigonometric equation for analytic reduction and geometric inspection of a robot’s links. However, the existence of optimal solution is not always guaranteed especially, when a high number of links is needed for spatial navigation.

In numerical approaches, relationships between Cartesian and joint space are iteratively optimized to derive a possible set of joint angles that can place an end-effector at desired positions in the workspace. Earlier studies were carried out based on linear approximations of actual motion in the form of Jacobian methods [23, 24]. For instance, Borboni et al. [25] evaluated pseudo-inverse matrix of a kinematic redundant serial-link robot developed for online pipe cutting in a continuous process of production, while Yazdani et al. [26] investigated fault-tolerant control of redundant serial manipulators with pseudo-inverse of the Jacobian. These might be unsuitable for medical procedure as several iterations are required to compute accurate joint angles for a given position. Wang and Chen [27] proposed cyclic coordinate descent (CCD) which became a popular method for solving IK. Recently, Aristidou and Lasenby [28] presented a forward and backward reaching IK (FABRIK) method for solving the kinematics of articulated models in computer animation. Both heuristic iterative approaches require low computational cost unlike Jacobian methods. However, they perform poorly in complex 3D models and only converge better around the planes.

Limitations in the IK methods above greatly affect transitioning of most existing snake-like robotic designs for real clinical purposes. ViaCath endoluminal system by Hansen Medical (Norwood, MA, USA) has been one of the few single-port articulated robots commercially available for laparoscopic surgery [29]. Experimental trials with the robotic tool, which comprises of flexible shaft and articulated tip with end-effector, have revealed kinematic control as its major limitation. Recently, Titan Medical Inc. (Toronto, ON, Canada) reported to have completed alpha prototype of a single incision abdominal surgical robotic system with single-use replaceable tips. The complete teleoperated system is expected to be commercialized upon FDA approval. Accurate and real-time IK method to snake-like robotic system has being a major factor hindering its wide acceptance into medical surgery; hence, this still remains a major research focus. By modeling, the problem can be viewed as solving nonlinear relationships between Cartesian and joint spaces with little dependencies. Approximations based on soft computing methods like fuzzy logic [30], neural networks [31, 32], and evolutionary optimization [33] have been proposed as alternatives to conventional IK approaches. However, the artificial intelligent methods require training adaptive algorithms for learning implicit relationships between the Cartesian and joint spaces of a particular robot. Moreover, learning periods require high storage, and the prediction error is uncontrollable.

Specifically, solving IK in robotics depends on design mechanisms of individual robot. Most existing snake-like robotic designs are not capable of accessing hidden abdominal organs in an accurate and timely manner. A common problem with existing designs is spatial inflexibility, that is, inability of a robot to achieve 3D curvy movements fitted for cluttered and confined paths in human body. Moreover, kinematic solutions for such robotic designs have reduced manipulable areas. This makes it is difficult to apply efficient avoidance schemes which could enhance effective interaction of the robot with other organs in a workspace. An IK method with admissible error offset, less computational time, and effective obstacle avoidance strategy is considered as been robust for spatial snake-like robots designed for MIS. Fortuitously, adapting biomorphic serpentine motion of natural snakes into such robots can simplify their IK model. By doing this, links of the robotic model are divided into several modules, and IK solution is defined for the lead-module only. The solution can be emulated by succeeding modules during translational movements.

The rest of this paper is arranged as follows. “Design of a flexible snake-like robot for radiosurgery” section presents the design of a flexible snake-like robot for radiotherapy of gastrointestinal cancer. “Proposed geometric method” section presents the non-iterative geometric method, proposed in this study, for solving IK of lead-modules in the snake-like robot. This section considers the flexible snake-like robot designed as a 2(n)-link manipulator and details of the proposed geometric method for cases of n = 1, and n = 2 are presented. Furthermore, the geometric method is extended for collision detection and avoidance with other organs in a workspace. “Results and evaluation of the proposed method” section presents implementation and evaluation details of the proposed method based on simulated and actual prototype of the snake-like robot. Finally, conclusion of the study and future works are presented in “Conclusion and future works”.

## Design of a flexible snake-like robot for radiosurgery

**Fig. 1:**
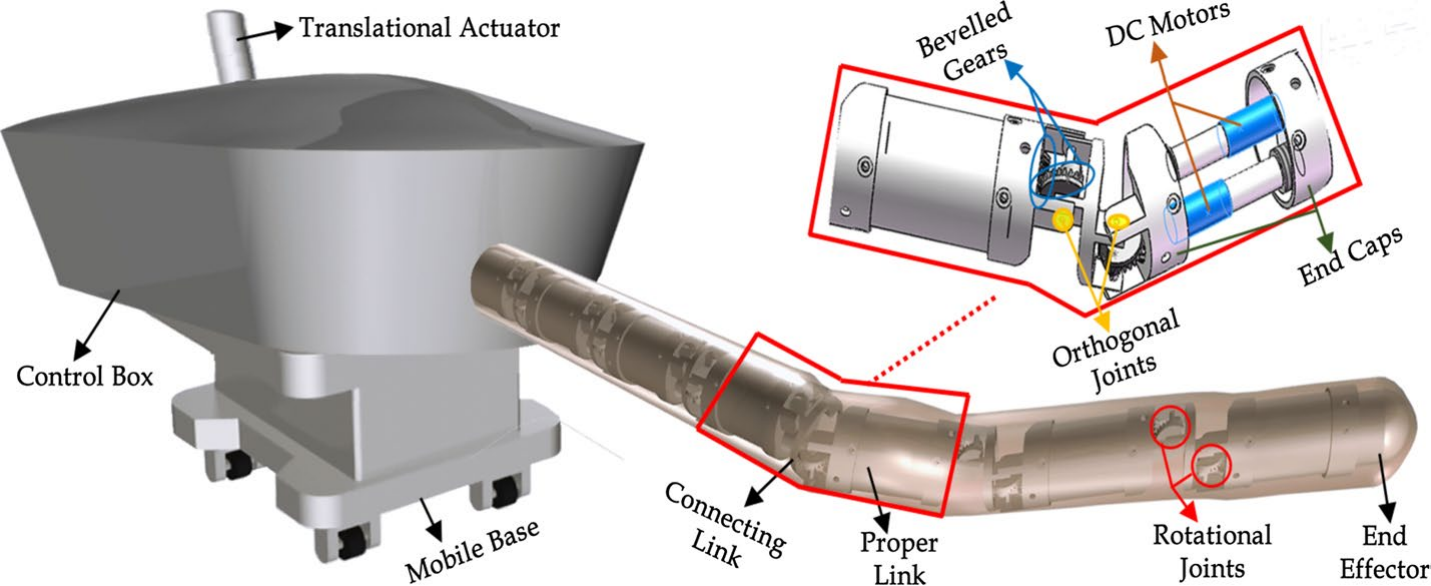
CAD model of the snake-like robotic model for radiotherapy

Figure 1 shows the CAD model of our design for the highly articulated robotic device for radiotherapy [14]. The design objective of the snake-like robot is to deliver radiotherapy dosage on cancerous cells around the gastrointestinal area in human abdomen. Instead of having invasions for excision of targeted cells, the robot is designed to go through natural orifices such as mouth, to the gastrointestinal tracts in human abdomen.

Unlike in Wilbert et al. [34] and Zhang et al. [35], the snake-like manipulator has single dexterous arm to perform radiosurgical operations. The radiosurgical robotic model was designed to exhibit serpentine movements of natural snakes during medical procedures. It has a translational actuator for driving the whole mechanism forward and backward while navigating through natural orifices, and a series of rotational actuators for controlling the end-effector. Each rotational actuator has a brushless DC motor made up of rotor with permanent magnets and stator with windings. Beveled gears with transmission ratio of 2:1 and reduction ratio of 625:1 are connected with the motors for speed and torque control, respectively.

Currently, this study focuses only on solving IK problem to control the rotational joints for a given target point. Rotational joints in the snake-like robot are used to connect its links as a modular structure. Each module has a pair of two links which are proper link and connecting link. Proper links are distinctively longer with a length of 53.12 mm, and has two caps fixed at both ends for connection. Each cap is used to house actuators for connection with paired connecting link. Each connecting link has a length of 11 mm, and basically used for interconnection between consecutive modules in the robot. Each link has a single DoF with its direction of rotation solely depending on the instantaneous angular value at the base of the link. The links within and between the modules of the robot are connected with a pair of orthogonally paired joints; thus, each consecutive joint has a joint twist value of ±*π*/2 rad. The DH parameters for robotic model with 1, 2, …, n modules are given in Table 1.

**Table 1:** DH parameters of snake-like model

*n*	Link id	Link length (a), (mm)	Twist angle (∝), (rad)	Link offset (d)	Joint angle (*θ*)
1	1	53.12	*π*/2	0	*θ*_1_
	2	11	−*π*/2	0	*θ*_2_
2	1	53.12	*π*/2	0	*θ*_1_
	2	11	−*π*/2	0	*θ*_2_
	3	53.12	*π*/2	0	*θ*_3_
	4	11	−*π*/2	0	*θ*_4_
*n*	1	53.12	*π*/2	0	*θ*_1_
	…	…	…	…	…
	2*n*	11	−*π*/2	0	*θ*_2*n*_

The lead-module of the robotic structure acts as its head. Just as natural snakes contract muscles in each segment of their body by thrusting from side to side while the head determines the direction of movement, initialization of motion in the modular mechanism starts with linear actuation of the lead-module followed by computation of IK required to position the end-effector at via- or target points. Previously, we applied optimized Monte Carlo method to achieve a precise IK solution for the snake-like robot with 12-DOFs [14]. In the study, forward kinematics of the robot configuration was used to compute possible poses in the robot’s workspace. The huge pose matrix was saved in a spreadsheet file and a search algorithm was used to select IK solution for given target points. The method has high response time, and fails to compute IK of target points that were not pre-stored in the spreadsheet.

Motivated by the above shortcomings, we will reduce the problem to solving the IK of the lead-module in this present study. Since serpentine movement could be realized with trans-rotational *follow*-*the*-*lead* motion using the translational actuator and the first n-rotational joints. While the former glides the snake robot forward and backward along X-axis, it is pertinent to determine appropriate joint angles that can lead the manipulator’s end-effector to a given target point. Hence, the translational actuator can advance all modules in the manipulator one after the other, and the IK solution of a *n*th module can be assumed by the *n*th + *1* module in the subsequent iteration. With this convention, it is sufficient to model IK solution just for links in the first module. This will not only reduce computation burden expected for the flexible articulated robots but also enhance its snake-like movement. Similarly, obstacle collision avoidance is an important area of the IK solution. This can necessitate alteration in a pre-defined trajectory of the snake-like robot during surgical operation. Hence, the proposed IK method is extended by utilizing redundancy found in the lead-module of the snake-like robot to detect and avoid collision with other organs in the workspace.

## Proposed geometric method

**Fig. 2:**
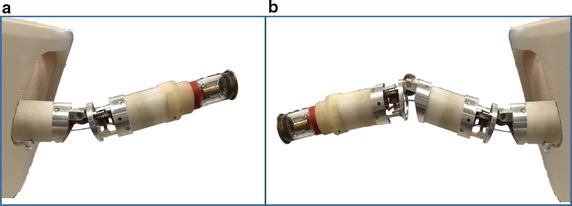
Spatial postures of the proposed snake-like robot. **a** Two-link module; **b** four-link module

The CAD model in Fig. 1 shows a modular robotic manipulator capable of snake-like movement with 2n links. We start the proposed geometric approach by computing IK solution for lead-modules with two links in Fig. 2a. This configuration may become trivially underactuated especially when one of the axial values in the target is close to zero. Hence, we extend the geometric method to solve IK of four-link module shown in Fig. 2b. This configuration is redundant, and existing methods cannot simultaneously solve its IK timely and accurately. Point virtualization is useful in solving IK of robotic models [36, 37], and collision avoidance with surrounding organs [38]. This serves as the basis of the geometric method we proposed in this study.

### Two-link module

The idea is to locate *n*-*1* virtual points (VPs) that are necessary to determine appropriate joint angles for a given target point in an *n*-*link* module. The angles were computed by setting of VPs in the Cartesian space of the workspace, and determination of angles between connected links. Details of both steps are explained in the following sub-sections.

#### Geometric setting of virtual points

**Fig. 3:**
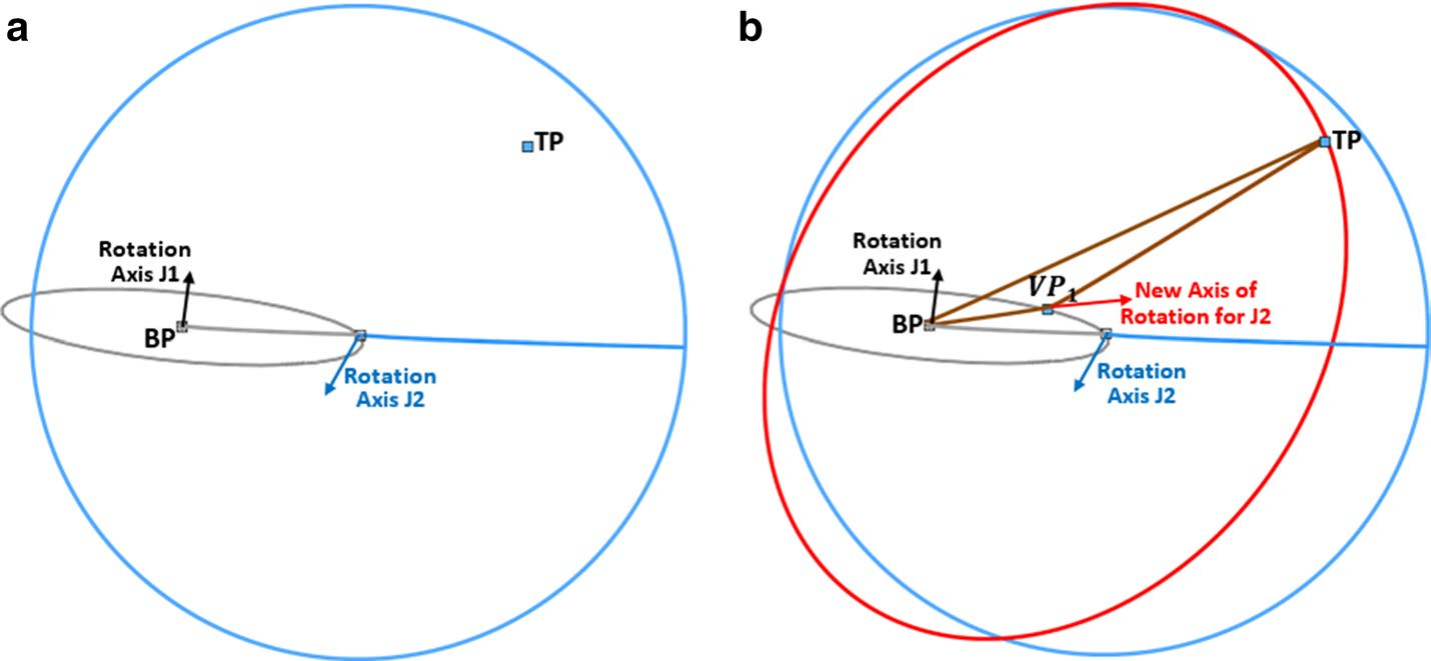
Location of virtual point between the base and target points. **a** Rotation axes of joints in the two-link module.   **b** Location of *VP*_1_ base on ​the rotation axes

A two-link module requires one VP to determine appropriate joint angles for a given target point. It should be pointed out that each link of any serial chain manipulator has a single axis of rotation, that is, it can only rotate in a unique plane. Hence, the VP should be located such that it coincides with tip of the first link and the base of second link at the same time. The distance between *VP*_1_ and the base point *BP* must be equal in length with the first link in the robotic model. Since the joints at both links are orthogonal to each other, rotation axis of the second link (the blue circles) will vary with respect to the joint value of first link as shown in Fig. 3a, b. To locate *VP*_1_, distance between *VP*_1_ and the target point (T*P*) must be equal to the length of second link in the manipulator. Hence, these constraints can be satisfied by computing an appropriate base angle (α) as in Eq. 1.

1 $$\alpha = cos^{ - 1} \left( {\frac{{L_{1}^{2} + \left| O \right|^{2} - L_{2}^{2} }}{{2*L_{{V_{1} }} *\left| O \right|}}} \right)$$

where *L*_1_ and *L*_2_ are the lengths of both links respectively, and |*O*| is the magnitude of the vector between the base and target points. Consequently, the third axial values of points *BP*_*Z*_ and $VP_{{1_{Z} }}$ are similar since the first link rotates around the default *z*-axis. Hence, coordinates of *VP*_1_ can be determined using Eq. 2.

2 $$VP_{1} = \left[ {\begin{array}{*{20}c} { \frac{{x + b\left( {\Delta x\cos \left( \alpha \right) - \Delta y \sin \left( \alpha \right)} \right)}}{{\sqrt {\Delta x^{2} + \Delta y^{2} } }},} & { \frac{{y + b\left( {\Delta y\cos \left( \alpha \right) + \Delta x \sin \left( \alpha \right)} \right)}}{{\sqrt {\Delta x^{2} + \Delta y^{2} } }},} & z \\ \end{array} } \right]$$

where $\left( {x, y, z} \right)$ are axial values of *BP*, and ($\Delta x$, $\Delta y$) are differences between the coordinates of *BP* and *TP*.

#### Computation of joint angles base on virtual points

**Fig. 4:**
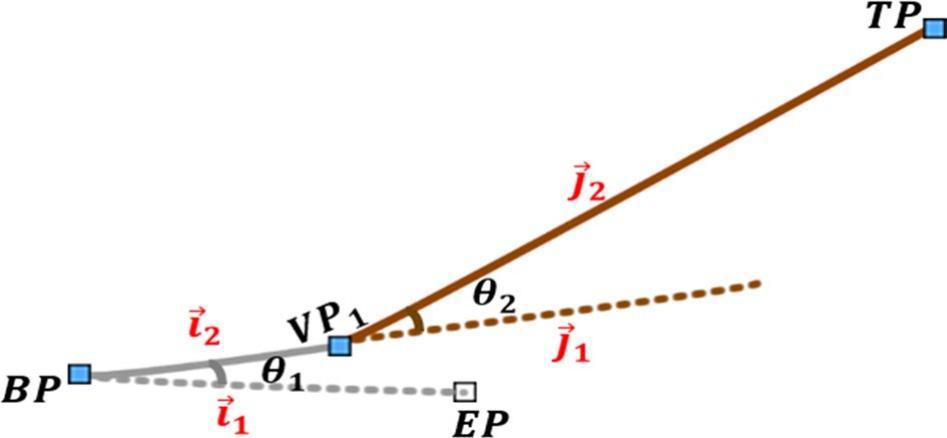
Joint angle computation in two-link module

Once an appropriate point is geometrically set as *VP*_1_, angles at both joints of the two-link module is obtained by vectorizing the known Cartesian points. Actually, we can make two vectors with the three known points $\left( {BP, \;VP_{1} ,\; TP} \right)$, however to determine the joint angles that connect the two links to their bases, an extra point (*EP*), which is the default coordinates of the tip of first link, is assumed as shown in Fig. 4.

Assuming $\left[ {\vec{i}_{1} , \vec{i}_{2} } \right]$ and $\left[ {\vec{j}_{1} , \vec{j}_{2} } \right]$ are equidistant vectors representing the initial and final poses of the two links respectively, the joint angles $\left( {\theta_{1} , \theta_{2} } \right)$ are calculated using the geometry of cross and dot products [39] represented as ⊗ and $\odot$, respectively, in Eq. 3.

3 $$\begin{array}{*{20}c} {\begin{array}{*{20}c} {\theta_{1} = atan2\left( {\vec{i}_{1} \otimes \vec{i}_{2} , \vec{i}_{1} \odot \vec{i}_{2} } \right)} \\ \\ \end{array} } \\ {\theta_{2} = atan2\left( {\vec{j}_{1} \otimes \vec{j}_{2} , \vec{j}_{1} \odot \vec{j}_{2} } \right)} \\ \end{array}$$

### Four-link module

In the last subsection, the proposed geometric method was modeled for two-link module. Exact and numerical approaches are also effective for solving IK problems of such configuration. Generally, both approaches perform better in modeling IK for serial robots with larger number of links provided that just one of the joints has twist value different from zero, or if the serial robot has an appropriate number of wrist offsets as in the case of most industrial robots. However, the approaches are not suitable for resolving IK of the snake-like model (in Fig. 2b) in a precise and timely manner. Hence, we will extend the geometric solution in “Two-link module” section to solve IK of four-link redundant module.

#### Determination of Cartesian coordinates of virtual points

Three VPs are necessary to determine appropriate joint angles for given target points in a *four*-*link* module, as explained in the case of ​two-link module. The VPs are classified either as mid-VP or connecting VP based on certain characteristics explained in this sub-section. Both VPs are used to determine possible coordinates of joints in the module such that the tip of the last link reaches a given via- or target point.

##### Mid-virtual point

To start with, the four-link module is sub-divided into two equidistant halves with a Mid-VP such that links in each half can be manipulated independently. Unlike in Sardana et al. [21], the proposed approach is applicable if all links of the manipulator have equal length, every two consecutive links have similar length pattern, or all links are distinctive in lengths. These three cases have different peculiarities but we will only focus on the second case as it is the case of the radiosurgical robot which this study is based on.

**Fig. 5:**
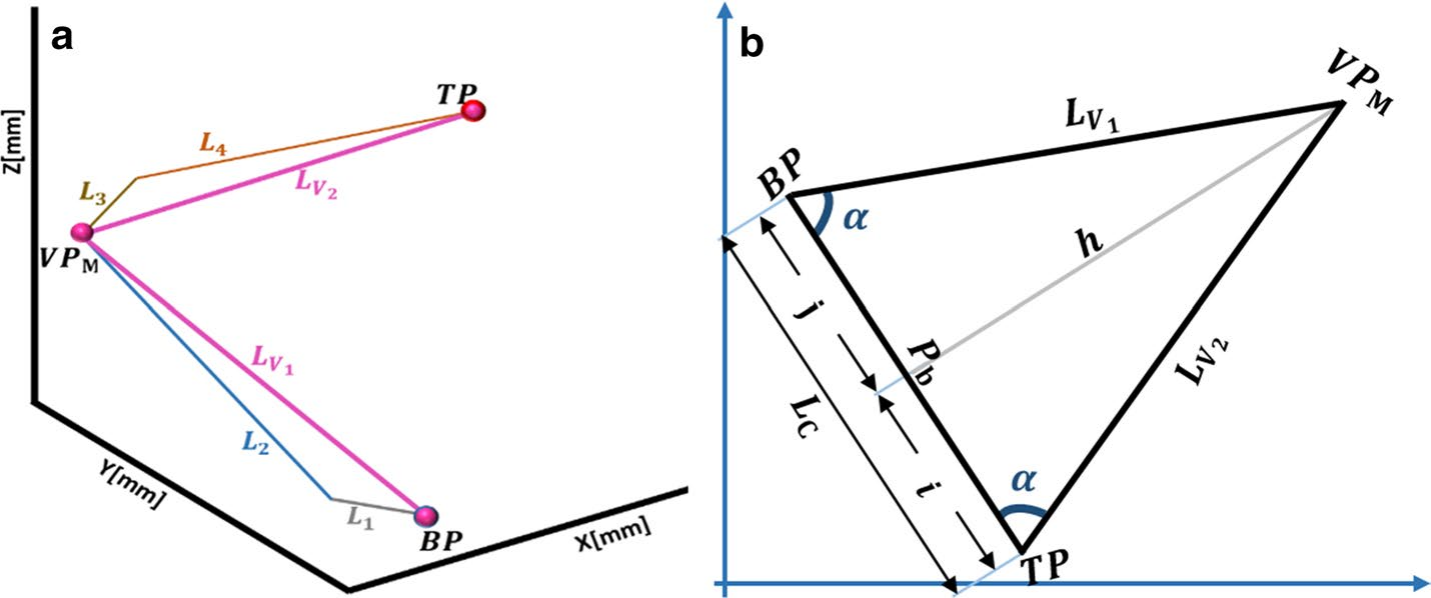
Virtualization of the four-link. **a** Determination of mid-virtual point. **b** Bisection of chord BP–TP

Consider the four-link module in Fig. 2b virtualized as an isosceles triangle shown in Fig. 5a, if the distance between the base and target points is a chord in a circle, a mid-virtual point (*VP*_*M*_) is chosen as center of the circle such that:

1. it extends with the lengths $L_{{V_{1} }}$ and $L_{{V_{2} }}$ from vertices BP and TP, respectively, in Fig. 5a, b and $L_{{V_{1} }} = L_{{V_{2} }}$. Hence, the two sides are said to be co-initial equidistant vectors from *VP*_*M*_;
2. magnitude of each equidistant vectors are greater than lengths of individual links of the robot, that is: $\left\{ {L_{{V_{1} }} , L_{{V_{2} }} } \right\} > L_{j}$ ∀*j* = 1, …, 4; and satisfies the triangular inequality rules: $L_{{V_{1} }} < L_{1} + L_{2}$ and $L_{{V_{2} }} < L_{3} + L_{4}$. These are explained graphically in Fig. 5a.
3. a line connecting *VP*_*M*_ to chord *BP*–*TP* perpendicularly bisects the chord at mid-point as shown in the planar view of Fig. 5b;

With a chosen *VP*_*M*_ satisfying the above conditions, Cartesian coordinates of the mid VP depends on the base angle (*α*) in the isosceles triangle in Fig. 5b. *α* is calculated using the Cosine expression in Eq. 4.

4 $$\alpha = cos^{ - 1} \left( {\frac{{L_{c}^{2} }}{{L_{c} *\left( {L_{{V_{1} }} + L_{{V_{2} }} } \right)}}} \right)$$

If correctly chosen, *VP*_*M*_ must be perpendicular to chord (*BP*–*TP*) at the mid-plane. Hence, Cartesian coordinates of the mid-plane, *P*_*b*_, is calculated using Eq. 5.

5 $$p_{b} = \frac{{ L_{{V_{1} }} }}{{ L_{{V_{1} }} + L_{{V_{2} }} }}* \overrightarrow {BT}$$

where $\overrightarrow {BT}$ is a spatial vector with initial and terminal points at *BP* and *TP* respectively with a magnitude of *L*_*c*_. Hence, the spatial coordinates of *VP*_*M*_ is parameterized by assuming a unit vector, $\vec{e}$, in the direction of $\overrightarrow {BT}$ from the base point, *BP*, such that the vectors: $\vec{e}$ and $\overrightarrow {BT}$ are linearly independent. If vectors $\vec{u}$ and $\vec{v}$ are normalized vectors formed by the cross products $\vec{u} = \left( {\vec{e} \otimes \overrightarrow {BT} } \right)$ and $\vec{v} = \left( {\overrightarrow {BT} \otimes \left( {\overrightarrow {BT} \otimes \vec{e}} \right)} \right)$ which are perpendicular to each other, and both vectors are perpendicular to $\overrightarrow {BT}$ in the geometric space, the spatial coordinates of *VP*_*M*_ is calculated with Eq. 6 using the height of bisector to the chord (*BP*–*TP*) given by Eq. 7.

6 $$VP_{M} = P_{b} + h\left( {\frac{{\vec{v}}}{{\left| {\left| {\vec{v}} \right|} \right|}}cos\left( \alpha \right) + \frac{{\vec{u}}}{{\left| {\left| {\vec{u}} \right|} \right|}}sin\left( \alpha \right)} \right)$$

7 $$h = \sqrt {\frac{{\left( {L_{{V_{1} }} + L_{{V_{2} }} } \right)^{2} - L_{c}^{2} }}{4}}$$

Alternatively, Eq. 6 can be simplified as given in Eqs. 8 and 9. That is, since coordinates of the base point $\left( {BP_{x} , BP_{y} , BP_{z} } \right)$ and $L_{{V_{1} }}$ are known, the axial values of *VP*_*M*_ in Eq. 6 can be calculated as ($x^{\prime}, y^{\prime}, z^{\prime}$) in Eq. 8.

8 $$\begin{array}{*{20}c} {x^{\prime} = BP_{x} + L_{{V_{1} }} *v_{x} } \\ {y^{\prime} = BP_{y} + L_{{V_{1} }} *v_{y} } \\ {z^{\prime} = BP_{z} + L_{{V_{1} }} *v_{z} } \\ \end{array}$$

where *v*_*x*_, *v*_*y*_, and *v*_*z*_ are the coordinates of a unit vector $\vec{v}$ defined in the direction of *VP*_M_ with Eq. 9.

9 $$\left[ {v_{x} , v_{y} , v_{z} } \right] = \left[ {\frac{\Delta xcos\left( \alpha \right) - \Delta y sin\left( \alpha \right)}{{\sqrt {\Delta x^{2} + \Delta y^{2} + \Delta z^{2} } }}, \;\frac{\Delta ycos\left( \alpha \right) + \Delta x sin\left( \alpha \right)}{{\sqrt {\Delta x^{2} + \Delta y^{2} + \Delta z^{2} } }}, \;\frac{\Delta zcos\left( \alpha \right)}{{\sqrt {\Delta x^{2} + \Delta y^{2} + \Delta z^{2} } }} } \right]$$

where $\Delta x$, $\Delta y,$ and $\Delta z$ are differences between the respective axial values of the base and target points.

##### Connecting virtual points

**Fig. 6:**
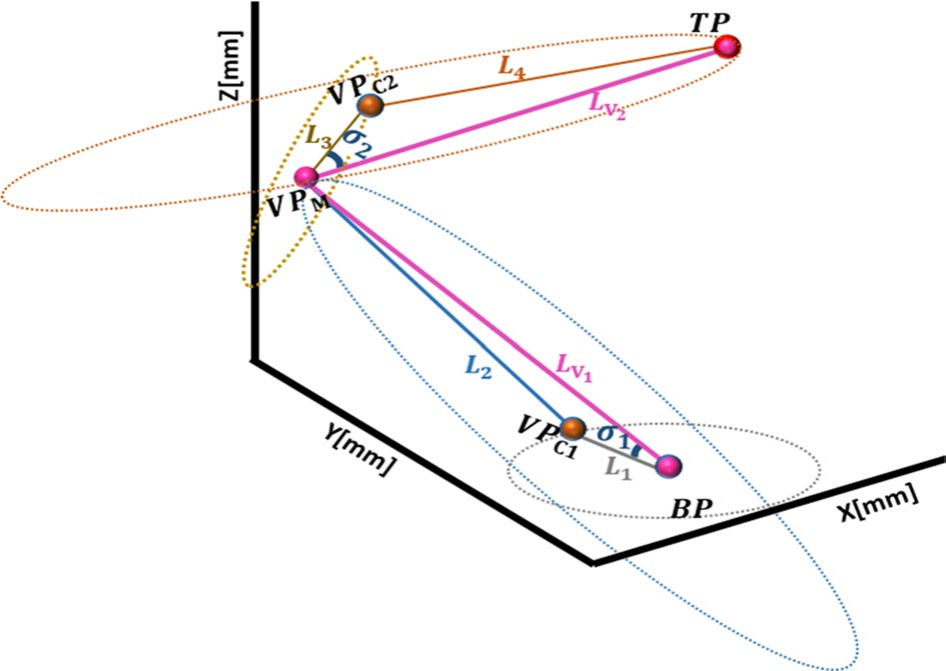
Connecting VPs in the rotation axes of four-link module

IK solution of the four-link module is complex and requires analysis of each link in distinct rotational axes of the geometric space. Exceptions to this are cases when the angles at first joint and third joint are zero, that is: *θ*_1_ = 0 and *θ*_3_ = 0; or when the angles at second and fourth joint are zero that is: *θ*_2_ = 0 and *θ*_4_ = 0. In both cases, the snake model works with only two-DoF and operates in planar configuration. In other cases, the complexity of the four-link module is reduced by defining a common point which can connect the links in each part formed by the Mid-VP. These points are regarded as the connecting virtual points (*VP*_*C*_), and are defined with respect to the Mid-VP (*VP*_*M*_) and the given target (*TP*) as shown in Fig. 6. Since each link has distinct rotational axis, as shown in Fig. 6, the two connecting virtual points are defined with respect to *VP*_*M*_. To define *VP*_*C*1_, *VP*_*M*_ is considered as target point with *BP* (in Fig. 6) as its base. However, *VP*_*M*_ is taken as the base point in the case of *VP*_*C*2_, and *TP* (in Fig. 6) being the target.

Rotations at first joint in each part of the module causes the connecting VPs to be in similar plane with their respective bases while rotation at the second joints in each part causes spatial deflection of the tip of the second link towards their respective targets. Hence, the *z*-axial values of bases and connecting VP in each part of the module are equal, and however, different from that of the target points. As a result, each link of the robot is capable of having different orientations. To locate *VP*_*C*1_ and *VP*_*C*2_ in Fig. 6, we need to compute their respective angles of elevation *σ*_1_ and *σ*_2_ in the sub-modules. Fortunately, the lengths of all sides are known, so we can apply the Cosine rule in Eqs. 10a and 10b to determine base angles: *σ*_1_ and *σ*_2_; with which the Cartesian coordinates of the connecting VPs are eventually computed in Eqs. 11 and 12.

10a $$\sigma_{1} = cos^{ - 1} \left( {\frac{{L_{{V_{1} }}^{2} + L_{1}^{2} - L_{2}^{2} }}{{2*L_{{V_{1} }} *L_{1} }}} \right)$$

10b $$\sigma_{2} = cos^{ - 1} \left( {\frac{{L_{{V_{2} }}^{2} + L_{3}^{2} - L_{4}^{2} }}{{2*L_{{V_{2} }} *L_{3} }}} \right)$$

11 $$VP_{C1} = \left[ {\begin{array}{*{20}c} { \frac{{BP_{x} + L_{1} \left( {\Delta x_{1} cos\left( {\sigma_{1} } \right) - \Delta y_{1} sin\left( {\sigma_{1} } \right)} \right)}}{{\sqrt {\Delta x_{1}^{2} + \Delta y_{1}^{2} } }},} & { \frac{{BP_{y} + L_{1} \left( {\Delta y_{1} cos\left( {\sigma_{1} } \right) + \Delta x_{1} sin\left( {\sigma_{1} } \right)} \right)}}{{\sqrt {\Delta x_{1}^{2} + \Delta y_{1}^{2} } }},} & {BP_{Z} } \\ \end{array} } \right]$$

12 $$VP_{C2} = \left[ {\begin{array}{*{20}c} { \frac{{VP_{Mx} + L_{3} \left( {\Delta x_{2} cos\left( {\sigma_{2} } \right) - \Delta y_{2} sin\left( {\sigma_{2} } \right)} \right)}}{{\sqrt {\Delta x_{2}^{2} + \Delta y_{2}^{2} } }},} & { \frac{{VP_{My} + L_{3} \left( {\Delta y_{2} cos\left( {\sigma_{2} } \right) + \Delta x_{2} sin\left( {\sigma_{2} } \right)} \right)}}{{\sqrt {\Delta x_{2}^{2} + \Delta y_{2}^{2} } }},} & {VP_{MZ} } \\ \end{array} } \right]$$

where ($\Delta x_{1}$, $\Delta y_{1}$) and ($\Delta x_{2}$, $\Delta y_{2}$) are the differences between the *x*, *y* axial values in the coordinates of points *BP*–*VP*_*M*_ and points *VP*_*M*_–*TP*, respectively.

#### Computation of robot’s joint angles base on virtual points

**Fig. 7:**
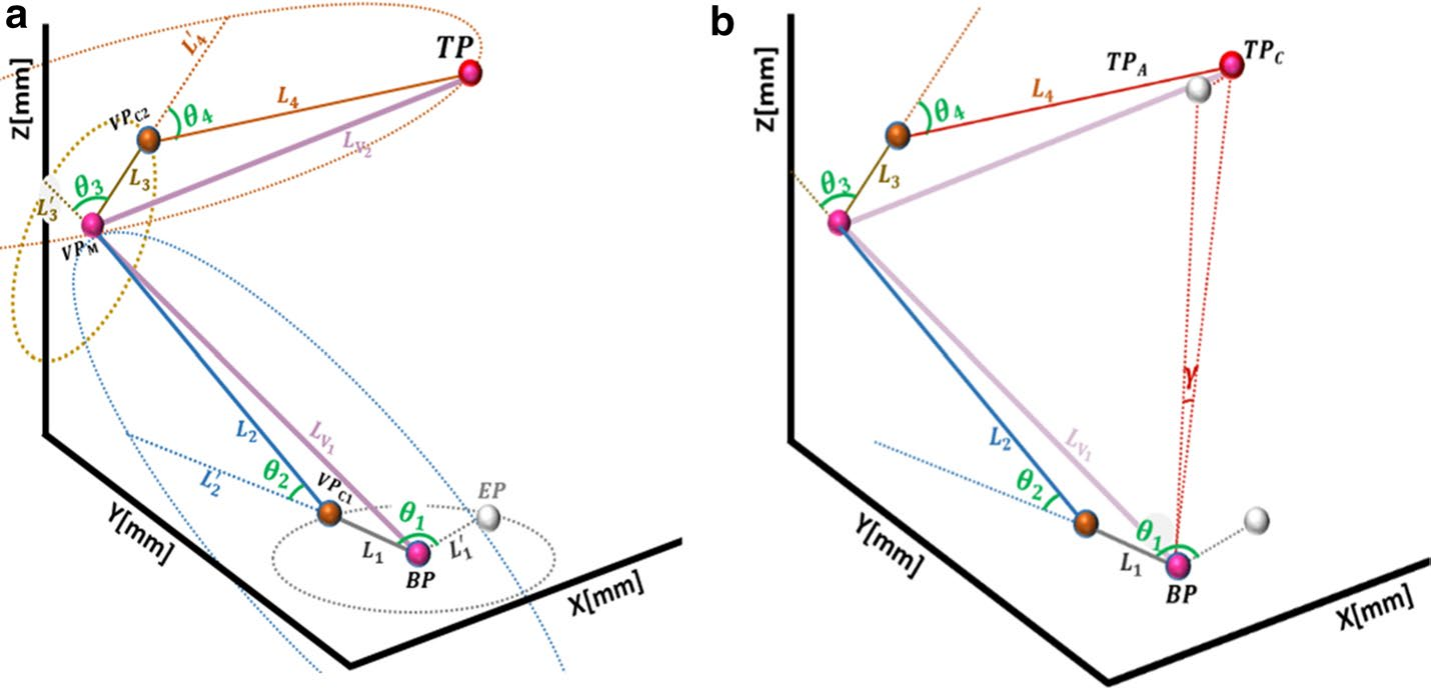
Vectorization of known points ($EP, BP, VP_{{C_{1} }} , VP_{M} , VP_{{C_{2} }}$ and *TP*) for joint angles in four-link module. **a** Computation of individual joint angles. **b** Error minimization from the base joint

In the last sub-sections, we have parameterized coordinates of the VPs needed to compute the joint angles with which a four-link module can use to arrive at given target point. The angular value at each joint is derived by vectorizing the Cartesian coordinates of the VPs obtained in the last sub-section. Alongside the base point, target point, and the three VPs, an extra point (*EP*), which is the initial pose of first link, is assumed as shown in Fig. 7a. Finally, the joint angles are computed using Eq. 13.

13 $$\theta_{k} = atan2\left( {\overrightarrow {{L_{k} }} \otimes \overrightarrow {{L_{k}^{{\prime }} }} , \overrightarrow {{L_{k} }} \odot \overrightarrow {{L_{k}^{{\prime }} }} } \right)$$

where *k* = 1, 2, 3, 4, and $\overrightarrow {{L_{k} }}$ and $\overrightarrow {{L_{k}^{'} }}$ are equidistant vectors representing the respective initial and final poses of the *k*th link used to compute the *k*th joint angle.

In the first condition, change in z-axial value ($\Delta z$) is zero if the rotation produced by second link does not cause elevation in the z-axis with respect to previous orientation. Before computing *θ*_3_, forward kinematics is employed to determine the actual position of *VP*_*M*_ based on joint angles *θ*_1_ and *θ*_2_. This is done as link transformation ${}_{ }^{i - 1} T_{i}$ from base point (*EP*) to *VP*_*M*_, with the standard DH convention in Eq. 14.

14 $${}^{i - 1}T_{i} = \left[ {\begin{array}{*{20}c} {cos \theta_{i} } & \quad { - sin \theta_{i} cos \alpha_{i} } & \quad {sin \theta_{i} sin \alpha_{i} } & \quad {\alpha_{i} cos \theta_{i} } \\ {sin \theta_{i} } & \quad {cos \theta_{i} cos \alpha_{i} } & \quad { - cos \theta_{i} sin \alpha_{i} } & \quad {\alpha_{i} sin \theta_{i} } \\ 0 & \quad {sin \alpha_{i} } & \quad {cos \alpha_{i} } & \quad {d_{i} } \\ 0 \quad & \quad 0 & \quad 0 & \quad 1 \\ \end{array} } \right]$$

Any change in the Cartesian coordinates of *VP*_*M*_ requires $VP_{C2}$ in Fig. 7 to be recalculated before the remaining angles $\left[ {\theta_{3} , \theta_{4} } \right]$ are computed. As explained in Wang et al. [40], the workspace of a robotic model is, cumulatively, the accessible area that can be referenced by the end effector. Since both sub-modules operate together in a single workspace and the consecutive joints have orthogonal relationship, there is possibility of position offset between the computed and actual target points. Possible error value was minimized with Eq. 15. This updates the value of *θ*_1_ with respect to the target position, as shown in Fig. 7b.

15 $$\theta_{1}^{{\prime }} = \theta_{1} \pm \gamma$$

where *γ* is the angular displacement by using the inverse of sine function. It should be noted that, this is only applicable when the computed target point, verified with *fkine* $\left( {\theta_{1}^{{\prime }} , \theta_{2} ,\theta_{3} , \theta_{4} } \right)$, is closer to the actual target point than *fkine* $\left( {\theta_{1} , \theta_{2} ,\theta_{3} , \theta_{4} } \right)$. This helps to obtain the best angle-set for given target point.

### Safety mechanism of the proposed IK method

Aside precise control and fast response, safety of an IK method is also important for effective interaction of surgical robots with non-targeted organs along a given trajectory. An avoidance strategy is added to the proposed geometric IK method for validating the VPs computed in “Two-link module” and “Four-link module” sections.

#### Collision detection

The proposed IK method is enriched with a function that avoids collision of the spatial snake-like robot with organs around a given trajectory in the manipulable area of the robot’s workspace. The first step is to check for possibility of collision between a link of the robot and such surrounding organs. For instance, if a via-point along a given trajectory is the target point (*TP*) set as input into the proposed IK method, collision with organs is analyzed based on individual rotation axis of each link in the robot. The VPs that are required to position the end-effector at *TP* are obtained as explained in “Four-link module” section. Thereafter, each link is circumscribed in a circle with radius equal to length of the link. Similarly, the organs in the manipulable area are circumscribed, uniquely, in different spheres. Assuming $\left[ {l_{X}^{i} , l_{Y}^{i} , l_{Z}^{i} } \right]$ and $\left[ {k_{X} , k_{Y} , k_{Z} } \right]$ are the center points of circumscription for *l*th link and *k*th non-targeted organ in the manipulable area, respectively; probability of collision between the link and the organ is checked with Eq. 16.

16 $${\text{PoC}}_{i - k} = \left\{ {\begin{array}{ll} {if \left( {\left( {\sqrt {\left( {k_{X} - l_{X}^{i} } \right)^{2} + \left( {k_{Y} - l_{Y}^{i} } \right)^{2} + \left( {k_{Z} - l_{Z}^{i} } \right)^{2} } } \right) - r_{i} } \right) \le d_{k} } & \quad 1 \\ & \\ {else} & \quad 0 \\ \end{array} } \right.$$

where PoC_*i*–*k*_ is the probability of collision; *r*_*i*_ and *d*_*k*_ are the radii of circular and spherical circumscription of the *l*th link and *k*th obstacle in the manipulable area, respectively.

#### Collision avoidance

This is an important step used to validate the aptness of VPs obtained in “Two-link module” and “Four-link module” sections for a given *TP*. Once the probability of collision between any of the links in snake-like robot and an organ around a given trajectory in the manipulable area of the workspace is set as one in Eq. 16, it is pertinent to find alternative joint configurations that can position the end-effector at *TP*. Thus, the initial VPs computed for the links could cause damage to the organ and should therefore be avoided. For the two-link module, only one *VP*_1_ exists for all target points reachable by the configuration. This is due to under-actuation in the Cartesian space and joint space of the configuration. It can be seen that any slight change in axial values of *VP*_1_ will deflect the end-effector from *TP* in Fig. 3b. This vindicates inexistence of two *VP*_1_ for a single target point in the under-actuated module.

Contrarily, the four-link module is redundant with an extra link and thereby gives room for more than one joint configuration for most target points. For an initial set of VPs, if the probability of collision of any of the links with an organ in the manipulable area is greater than zero, a collision avoidance strategy is implemented. This is done by modifying base angle (*α*) in Eq. 4 so that an alternative location of *VP*_*M*_ in Fig. 5 can be computed. The base angle is redefined as Eq. 17 and new coordinates of the mid VP is recomputed.

17 $$\alpha = - \left( {cos^{ - 1} \left( {\frac{{L_{c}^{2} }}{{L_{c} *\left( {L_{{V_{1} }} + L_{{V_{2} }} } \right)}}} \right)} \right)$$

Similarly, several locations of *VP*_*M*_ can be gotten by varying length of the bisector of chord *BP*–*TP*. Each new *VP*_*M*_ gives a unique IK solution which can be used to avoid collision with surrounding organs in the workspace. These procedure are utilized for safe interaction of the robot with other organs in robot’s manipulable area.

## Results and evaluation of the proposed method

The proposed method was implemented on simulated and actual prototype of the snake-like robot. Results of the proposed method and evaluation against commonly used methods are presented in this section.

### Implementations and results

The proposed method was tested in Matlab simulation and the current prototype of the snake-like. Models for two-link and four-link modules were simulated in Matlab 8.3 (R2014a) using Robotics Toolbox [41]. This study was carried out on a Lenovo M4380 desktop Computer with Intel^®^ duo processor of Core i3-3420 (2.40 GHz each), and a 2 GB RAM. The DH parameters of both modular configuration are cases of *n* = 1 and *n* = 2, respectively, in Table 1. It should be noted that the four-link module is capable of spatial rotations in four orthogonal planes without offset in any joint. To the best of our knowledge, there exists no single method that has solved the IK of such modular configuration with both with high accuracy and short computational time, simultaneously.

#### Simulation results from arbitrary points

Following the procedures of the geometric method proposed in this study, the computation of virtual points necessary for both two-link and four-link modules to reach target points in their respective workspaces are given in Table 2. The base point is always positioned at the origin (0, 0, 0) in both configurations while coordinates of some reachable points are set as the target. Only one VP is needed in the two-link configuration as shown in the first four rows of Table 2. It can be seen that the values at z axis of base point and *VP*_C1_ are equal and similarly for *VP*_*M*_ and *VP*_C2_. The value of $V_{{{\text{L}}_{i = 1,2} }}$ was rationally fixed at 64 mm in this simulation based on the conditions explained in “Determination of Cartesian coordinates of virtual points” section. The joint angles were computed based on Cartesian coordinates obtained for the derived VPs in Table 2, in addition with the Cartesian coordinates of the fixed base point (0, 0, 0); the target point which is the input to the geometric method.

**Table 2:** Computation of virtual points in the proposed geometric IK model

Id	Target position (mm)			VP_C1_ (mm)			VP_M_ (mm)			VP_C2_ (mm)		
	X	Y	Z	X	Y	Z	X	Y	Z	X	Y	Z
1^a^	35.895	47.082	22.319	6.669	8.477	0						
2^a^	11.162	32.623	−47.650	3.561	10.408	0						
3^a^	−20.562	−35.615	10.237	5.500	9.526	0						
4^a^	−46.787	−33.441	−25.663	−8.949	−6.396	0						
5^a^	−17.643	34.559	45.263	−5.002	9.797	0						
6	−23.033	44.716	16.960	−10.987	−0.538	0	−63.289	−3.097	8.924	−56.205	5.318	8.924
7	95.978	20.201	−7.583	6.804	8.643	0	39.576	50.276	−3.795	49.282	45.100	−3.795
8	−70.092	96.494	−13.310	−9.188	6.049	0	−53.205	35.030	−6.652	−56.120	45.637	−6.652
9	−40.512	21.263	−13.329	−9.490	−5.562	0	−54.933	−32.193	−6.889	−52.420	−21.484	−6.889
10	−102.447	−12.848	−7.496	−8.003	−7.5469	0	−46.553	−43.900	−3.751	−56.168	−38.558	−3.751
11	4.644	28.145	10.142	−10.124	4.302	0	−58.757	24.971	5.423	−47.770	25.521	5.423
12	−42.313	−35.407	21.584	−8.365	−7.142	0	−48.417	−41.340	−6.942	−47.039	−30.429	−6.942
13	124.266	−26.778	−10.907	10.932	−1.220	0	63.446	−7.083	−5.449	73.9107	−10.4715	−5.449
14	52.552	−106.386	11.648	8.161	−7.375	0	47.335	−42.777	5.823	48.234	−53.740	5.823
15	29.670	104.466	19.317	−2.813	10.634	0	−16.167	61.129	9.677	−8.174	68.686	9.677

^a^2-link-module

**Fig. 8:**
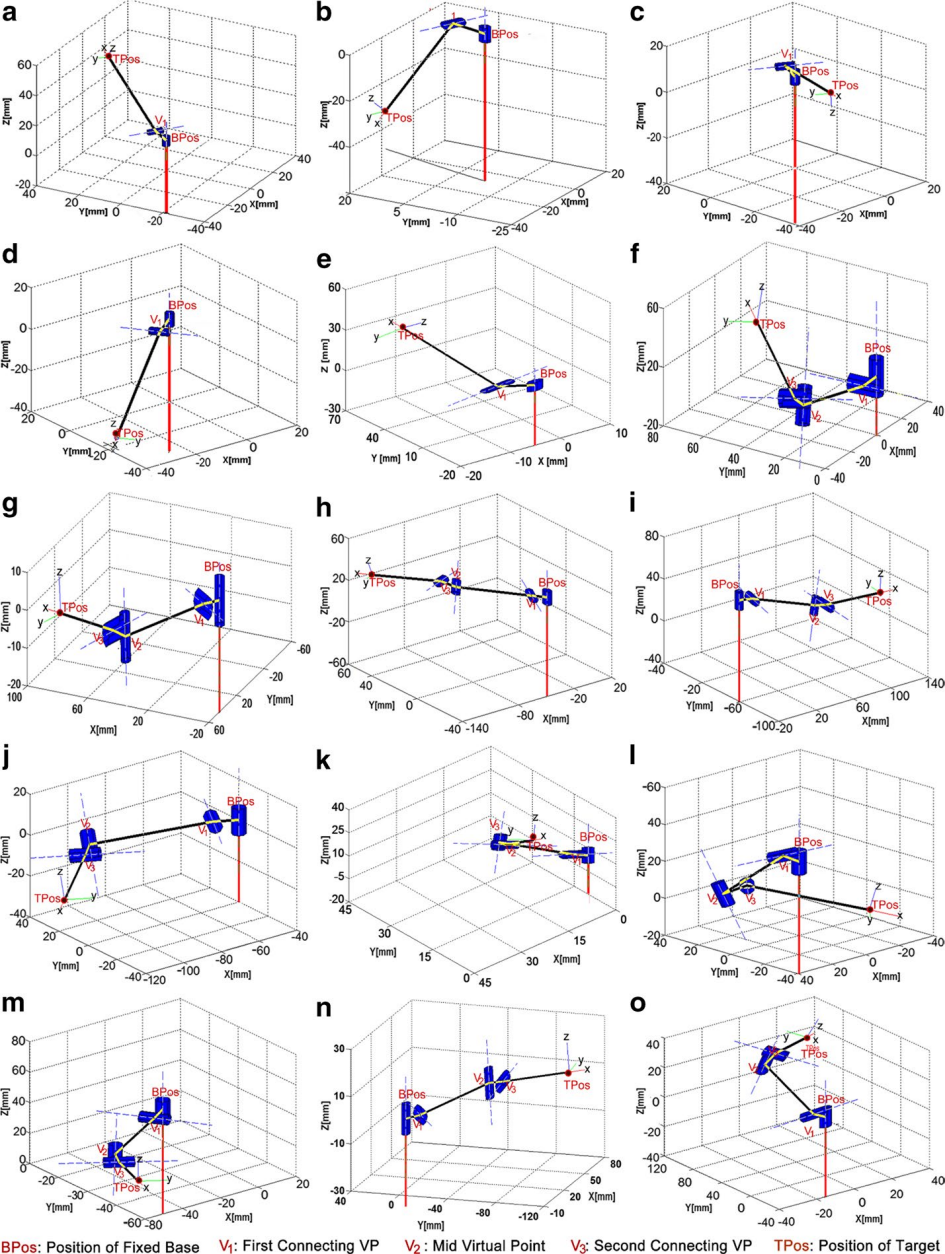
Simulation results for the arbitrary target points in Table 2. **a**–**o** ​Matlab plots for the 15 target points in Table 3

Coordinates of the extra point (EP) used for this simulation is (11, 0, 0). This is the default location of the tip of first link in the snake-like robot. The fifteen postures in Fig. 8 are the Matlab plots obtained when theta values computed by the proposed method are set as joint angular values in both configurations.

#### Simulation result from pre-defined path with obstacles

Implementation of the proposed IK method was also verified for safe interaction with organs around given paths in the workspace. For this purpose, boundary of an ellipsoidal shape was set as desired path, amidst several bodily organs represented as spherical objects with varying sizes in the workspace. Axial values of points along the ellipsoidal path were generated with Eq. (18), and set as targets of the robot, consecutively.

18 $$\left( {x - 30} \right)^{2} + \left( {y - 25} \right)^{2} + \left( {z - 55} \right)^{2} = 33$$

**Fig. 9:**
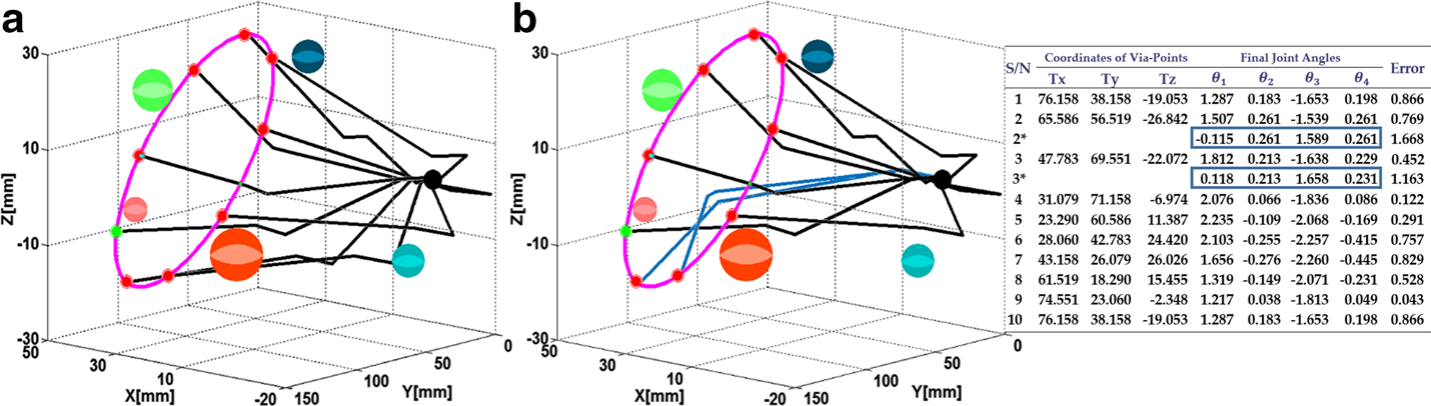
Interaction with surrounding organs. **a** Ordinary IK method. **b** IK with collision avoidance

The parameterized ellipsoidal path is made up of 100 spatial points out of which 10 via-points were evenly chosen and set as input into the proposed IK method. As shown in Fig. 9, the proposed method was able to resolve the kinematics based on the target points. The probability of collision was greater than zero for 4th link and 2nd link in transitions *P*_1_–*P*_2_ and *P*_2_–*P*_3_, respectively. In both cases, the proposed method used the alternative base angle to compute another set of VPs that could move the links through the via-points successfully without collision. The target points and corresponding final joint angles computed for the points are shown at right hand side of Fig. 9b.

#### Experimental results

**Fig. 10:**
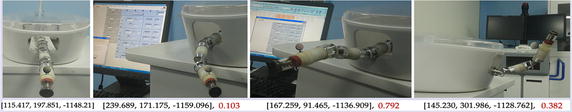
Final postures of actual robot for last three target points in Table 2

The proposed method was also tested on prototype of the snake-like robot. Currently, the robotic mechanism can only support four-link drive due to iterative prototyping methodology adopted for its design. The geometric IK method for four-link module was implemented graphically in LabVIEW^®^ (National Instruments). This programming environment was chosen because of its built-in support for NI CompactRIO (cRIO-9118) with FPGA reconfigurable chassis and digital I/O modules which were used for controlling motor drives at the robot’s joints. Suitability of the proposed method was tested with the actual robot using coordinates of the last three points in Table 2. The joint angles obtained from the geometric method were set at each joint of the robot, and position of tip of the last link was recorded. The current prototype of the snake-like robot does not feature a position sensor. To obtain its position in the world coordinate system, an NDI passive marker was fixed at tip of the robot’s last link, and its Cartesian location was recorded with a Polaris NDI tracking navigation system [Polaris Spectra, Northern Digital Inc. (NDI), Canada]. The default posture of the actual robot and final postures obtained from the last three target points in Table 2 are shown in Fig. 10a–d respectively. Location of the marker recorded for each target point is appended to the corresponding postures in Fig. 10.

It can be seen from Fig. 10 that the snake-like robot can achieved similar results as the simulated model. The final tip position in each figure is closely reached when the robot’s joints were rotated by angles calculated from the proposed IK method. Error offsets in final postures of the actual robot with respect to the simulated model are annotated with red in the figures. The low offset values further validate the proposed geometric approach.

### Model evaluation and discussions

Position accuracy and response time are two major metrics that are commonly used to evaluate IK methods in medical robotics. Basically, position accuracy is the euclidean distance between a given target point and the actual point reached by an IK method, while response time is the total execution time needed to solve IK problem for a single target point. The proposed method is validated with both metrics in this section.

#### Arbitrary points in workspace

First, we evaluated the proposed method based on error offsets in each of the plots in Fig. 8. We applied forward kinematics to determine the actual coordinates of last link’s tip when the joint angles computed by our proposed method were set as input. The error values are the differences between axial values of given targets and the corresponding actual points in each plot. The joint angles computed for each point, alongside actual points and error values are summarized in Table 3.

**Table 3:** Evaluation based on 15 arbitrary target points

Id	Target position (mm)			Actual tip point (mm)			Joint angles (rad)				Error values (mm)			*e* (mm)
	X	Y	Z	X	Y	Z	*θ*_1_	*θ*_2_	*θ*_3_	*θ*_4_	X	Y	Z	
1^a^	35.895	47.082	22.319	35.894	47.082	22.319	0.919	−0.434			−3.69^−4^	−4.85^−4^	−2.82^−4^	6.71^−4^
2^a^	11.162	32.623	−47.650	11.162	32.623	−47.649	1.241	1.113			−1.14^−4^	−3.34^−4^	7.16^−4^	7.98^−4^
3^a^	−20.562	−35.615	10.237	−20.562	−35.615	10.237	1.047	−2.948			1.14^−4^	1.98^−4^	−4.48^−5^	2.33^−4^
4^a^	−46.787	−33.441	−25.663	−46.787	−33.441	−25.663	−2.521	0.504			−1.77^−4^	−1.27^−4^	−1.20^−4^	2.49^−4^
5^a^	−17.643	34.559	45.263	−17.643	34.559	45.263	2.043	−1.020			−9.10^−5^	1.78^−4^	3.26^−4^	3.82^−4^
6	−23.033	44.716	16.960	−22.893	44.443	17.027	−3.075	−0.169	−2.306	−0.292	0.141	−0.274	0.067	0.315
7	95.978	20.201	−7.583	95.860	20.201	−7.581	0.907	0.072	−1.394	0.056	−0.118	0.000	0.002	0.118
8	−70.092	96.494	−13.310	−69.766	96.251	−13.143	2.518	−0.055	−0.663	0.361	0.327	−0.243	0.167	0.440
9	−40.512	21.263	−13.329	−40.436	21.237	−13.375	−2.424	0.131	−2.394	0.238	0.076	−0.026	−0.463	0.093
10	−102.447	−12.848	−7.496	−102.421	−12.852	−7.499	2.634	0.147	1.262	−0.060	0.026	−0.004	−0.003	0.027
11	4.644	28.145	10.142	4.502	28.553	10.194	2.7539	−0.102	−2.679	−0.201	−0.142	0.408	0.052	0.435
12	−42.313	−35.407	21.584	−41.716	−34.948	21.660	−1.324	−0.218	−2.205	−0.354	0.595	0.459	0.076	0.755
13	124.266	−26.778	−10.907	123.671	−26.808	−9.911	−0.333	0.159	0.239	−0.158	−0.596	−0.030	0.997	1.162
14	52.552	−106.386	11.648	52.513	−106.138	11.561	−0.735	−0.110	−0.760	−0.018	0.039	−0.248	0.087	0.266
15	29.670	104.466	19.317	29.723	103.418	20.022	1.765	−0.529	−1.020	0.500	0.054	−1.048	0.705	1.264

^a^2-link-module

Magnitude of the error values are given in the last column of Table 3. It can be observed that the error values obtained for the two-link module are always less than 1 µm. This is because the module has just two-links and its exact solution comprises of three unknown in two equations, hence, some dependencies can be established. However, the error values in the case of four-link module are comparatively large. These can be attributed to existence of differences in direction of rotation of each link. Irrespectively, tip of the last link is always very close to the target with maximum error of 1 mm except in rows 13 and 15 of Table 3.

#### Complete workspace evaluation

**Fig. 11:**
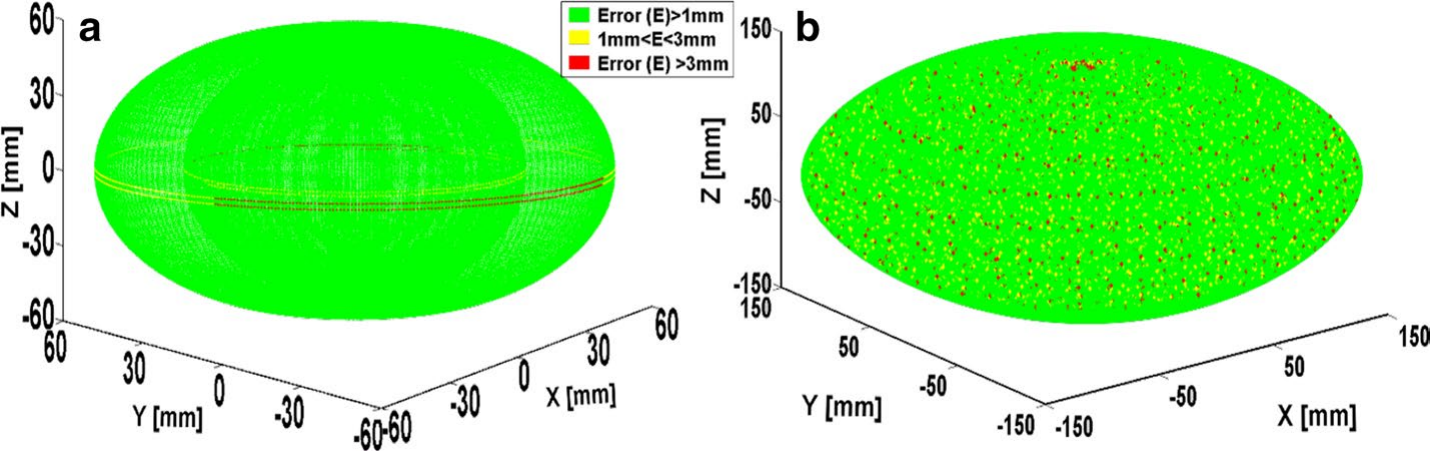
Evaluation of proposed method with workspaces of two- link (**a**) and four-link (**b**) modules

Robustness of the proposed geometric method is further checked by validating points that make up the complete workspace. By using 0.01425 and 0.3 rad as angular interval, workspaces for both two-link and four-link modules are generated with forward kinematics. As presented in Table 4, validation result shows the proposed method can determine 99.21% of points in the two-link’s workspace, 88.61% of points in the four-link’s workspace with error threshold value of 1 mm. The percentage accuracy of the latter also reached 99.50% when the error threshold was set as 3 mm. Nonetheless, there were still some points with position error values greater than 3 mm in both workspaces as shown in plots of Fig. 11.

**Table 4:** Error analysis of the proposed geometric method

Robot configuration	Angular interval (rad)	Total points in workspace	Points reached by link configuration	Percentage reachability (%)	Error threshold (mm)
Two links, 1 Twist	0.01425	194,481	192,939	99.21	1
Four links, 2 Twist	0.3		172,338	88.61	1
			193,507	99.49	3

Analyses of both workspaces show that, the percentage accuracy of the proposed geometric method is relatively high. Although, several points in both workspaces were still unreachable despite increasing the error threshold to 3 mm. Most of these points are found at the mid-horizontal boundaries of both workspaces where the DOFs in succeeding links cannot be fully utilized due to certain rotations that occurred at one or more preceding joint(s).

### Comparison with existing methods

Finally, the proposed method is compared with some existing methods used in solving IK problems. For this purpose, we implemented Jacobian Damped Least Squares (DLS), Jacobian Transpose, and Jacobian Damped Least Squares with Singular Value Decomposition (SVD-DLS), which are popularly used in robotics control. Details of these numerical approaches can be found in [24]. In addition, we implemented CCD [27] and fast iterative solver [28] which are geometric methods with iterative error minimization. We considered iterative methods only because, to the best of our knowledge, there is no exact approach for the proposed snake-like model at the time of this study.

A careful observation on Fig. 11a shows the effects of under-actuation of two-link module. Hence, we used the four-link model to evaluate all the six methods. To eliminate bias effects from the positive gain constants in the numerical methods, we set a unique value of 1 for both alpha and lambda. Also, we set a value of 500 as the maximum number of iterations to avoid endlessness in cases where such points are unreachable for any of the method. Since response time and position accuracy are major parameters needed for operation of robotic manipulators in assisted-radiosurgery, performances of the six methods were adjudged based on percentage accuracy, mean execution time, and average number of iteration of each method over 194,481 points that make up the workspace. Results of comparison are summarized in Table 5.

**Table 5:** Comparison of the six methods based on accuracy, execution time, and mean iteration

IK methods	Ranges of error magnitude (mm)			Execution time (s)			Average iteration
	*e* ≤ 1	1 < *e* ≤ 3	*e* > 3	*t* ≤ 0.1	0.1 < *t* ≤ 1	*t* > 1	
Proposed method	172,338	21,169	974	194,481	0	0	1
Jacobian SVD−DLS	193,514	211	756	729	187,437	6315	12.61 ± 23.7
Jacobian DLS	192,389	1139	953	8	187,679	6794	20.58 ± 67.8
Jacobian transpose	8103	10,361	176,417	0	4751	189,730	500
CCD	12,192	14,795	167,494	23	7617	186,841	379.46 ± 185.3
FABRIK	18,788	22,528	153,165	39,613	11,649	143,219	327.12 ± 217.5

**Fig. 12:**
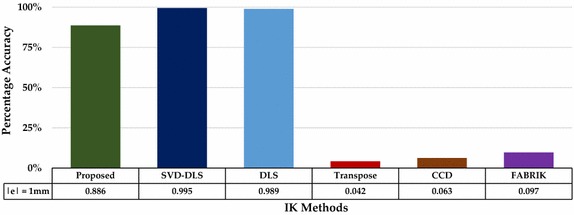
Percentage accuracy of the IK methods with error threshold of 1 mm

With an admissive error threshold of 1 mm, the percentage accuracies obtained for the six methods are shown in Fig. 12. It can be observed from bars of the plot that Jacobian SVD-DLS and Jacobian DLS can compute IK for target points in the four-link module workspace with percentage accuracies of 99.5 and 98.9%, respectively, while the proposed method has a percentage accuracy of 88.6%. Aside its accuracy, the proposed method has the least response time when compared with other methods.

Response time is the total execution time of an IK method for a single point-to-point movement. The proposed method has a maximum response time of 0.009 s, which is significantly close to zero in reaching each point in the four-link robot’s workspace.

**Fig. 13:**
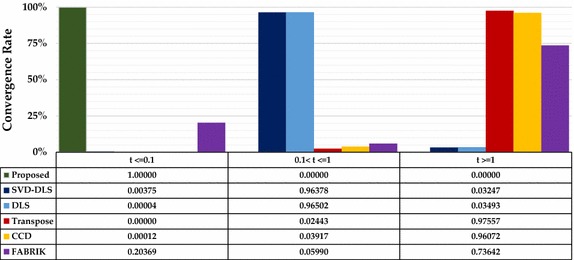
Percentage convergence of IK methods with respect to time (s)

As clearly indicated in Fig. 13, execution times of both SVD-DLS and DLS methods are higher than that of the proposed geometric method. Both methods have response time less than 0.1 s in approximately 0.004% of points in the whole workspace. Both methods (that is SVD-DLS and DLS) require mean execution times of 0.36 and 0.41 s, respectively, to compute the IK of a given target point. In their best cases, the two methods completed computation of IK for 96.5% of the points in the workspace at approximately 0.95 s. On the other hand, the proposed geometric method requires less than 0.01 s to compute IK of any point in the workspace. This is more than 30 times faster than both DLS and SVD-DLS, hence the proposed method has the shortest computational time when compared with other methods.

It is pertinent to emphasis that accuracy is an important factor in medical robotics. However, despite the high accuracies of SVD-DLS and DLS methods (reported in Table 5), the methods cannot be used for solving IK of such surgical robot as it will cause delay in a master–slave teleoperative system. A way to ensure trade-off between these two sensitive metrics is to widen the admissive error threshold for the sake of efficient run-time control. As reported for the four-link module, a careful analysis with special focus on accuracy shows that the proposed geometric method can compute IK of 99.49% of the workspace points with a maximum error of 3 mm. Hence, aside an extra merit of being computationally faster, setting the error threshold value as 3 mm makes the accuracy of the proposed method to meet up with both SVD-DLS and DLS methods; and it can be very applicable in radiosurgery.

The performances of the other three methods are not encouraging based on both metrics. FABRIK performed better than both CCD and transpose technique in terms of both metrics. For instance, it attained percentage accuracies of 9.7% at an error threshold value of 1 mm, and also converged in 20.34% when t ≤ 0.1 s. Also, CCD attained accuracies of 6.3% at an error threshold value of 1 mm. It performed better than Jacobian transpose which is very unstable. Usually, the three methods require more than 500 iterations to reach desired target points. This was confirmed by increasing the maximum iteration value to 1000 and, in fact, they only performed better when one of the axial values of target points is close to zero. Despite increasing the threshold value to 3 mm the three methods could only determine 9.5, 13.9, and 21.2% points accurately in the workspace with an average execution time of 4.78 s, 7.08 s, and 15.71 s, respectively. Although, the Jacobian methods have natural tendency to cope with joint complexities, however they converge very slowly. This can be attributed to the consistent orthogonal joints that connect consecutive links in the design of the proposed snake-like robot. Hence, the methods are quite unreasonable for the purpose of the snake-like robot proposed in this study.

## Conclusion and future works

In this study, a non-iterative geometric method has been proposed for solving IK of lead-module in spatially redundant snake-like robotic manipulator designed for radiosurgical operation. The modular robot is designed such that there are 2*n* links in each module and the direction of rotation of each link is orthogonal to the preceding one. This is quite complex for existing approaches; hence, the basic idea of the proposed method is to define *n*-1 VPs in the workspace of an *n*-link module. Positions of the VPs determine viable joint space (posture) needed to place tip of the last link at given targets in a workspace. Similarly, location of the mid-*VP* for a given point can be varied to compute multiple IK solutions. This is useful for collision avoidance and safe interaction between the snake-like robot and other organs in its workspace. In this paper, the geometric method is applied to solve IK of two-link and four-link modules which are capable of spatial navigations. Results from Matlab simulation prototype of the snake-like robot show that the proposed method is computationally inexpensive, fast and accurate. Collision avoidance strategy enriches the proposed method in switching between different joint configurations rather than interrupting movements of links in cluttered areas.

In the future, we will investigate on how to transfer IK solution of the lead-module to succeeding ones in a trans-rotational iterative process such that the manipulator can replicate snake motion. This work is part of a developmental robotic system for abdominal radiosurgery with minimal and no-invasion. This paper focuses on solving the kinematic problem of the robotic system. There is need to improve the IK method for automatic selection of optimal joint configuration on run-time. Furthermore, path planning, trajectory, and analysis of torque and force acting at each joint are vital for success of surgery in real clinical situations. Finally, dynamics of the actual robot shall be considered along with the IK method for minimal or no invasion experimentation trials in animal.

## Abbreviations

DH: Denavit–Hartenberg
IK: inverse kinematics
VP: virtual points
TP: target point
BP: base point
EP: extra point
DLS: damped least squares
SVD: singular value decomposition
CCD: cyclic coordinate descent
FABRIK: forward and backward reaching inverse kinematics
